# Determinants of Anemia in Pregnancy: Findings from the Ethiopian Health and Demographic Survey

**DOI:** 10.1155/2020/2902498

**Published:** 2020-06-05

**Authors:** Ataklti Gebretsadik Woldegebriel, Gebremedhin Gebregziabiher Gebrehiwot, Abraham Aregay Desta, Kiros Fenta Ajemu, Asfawosen Aregay Berhe, Tewolde Wubayehu Woldearegay, Nega Mamo Bezabih

**Affiliations:** ^1^Tigray Health Research Institute, Mekelle, Tigray, Ethiopia; ^2^Adigrate University, Mekelle, Tigray, Ethiopia

## Abstract

In Ethiopia, anemia during pregnancy is a major public health problem and affects both the mother's and their child's health. There is a scarcity of community-based evidence on determinants of anemia among pregnant women in the country. Therefore, this study aimed to assess the determinants of anemia among pregnant women in Ethiopia. *Method*. This study was based on the 2016 Ethiopian Demographic Health Survey (EDHS) that used a two-stage stratified cluster sampling technique. A cross-sectional study was conducted among 3080 pregnant women. Data analysis was done using STATA v.14. Variables with *P* value <0.05 in the bivariate analysis were candidates for the multivariable analysis to identify independent determinants of anemia among pregnant mothers. Odds ratios (OR) were calculated at 95% confidence interval (CI). *Results*. The overall prevalence of anemia among pregnant women was 41% of which 20% were moderately anemic, 18%, mildly anemic, and 3%, severely anemic. The following were significantly associated with anemia during pregnancy: an age of 30–39 years, receiving no education (AOR = 2.19; 95% CI 1.45, 2.49), belonging to the poorest wealth quintile (AOR = 1.29; 95% CI 1.22, 1.60), being a Muslim (AOR = 1.59; 95% CI 1.69, 2.65), number of house members being 4–6 (AOR = 1.44; 95% CI 1.05, 1.97), number of under-five children being two (AOR = 1.47; 95% CI 1.10, 1.97), head of the household being a female (AOR = 2.02; 95% CI 1.61, 2.54), current pregnancy wanted later (AOR = 1.75; 95% CI 1.23, 1.63), no terminated pregnancy (AOR = 1.49; 95% CI 1.15, 1.93), and an age of 13–17 years at the first sexual intercourse (AOR = 1.97; 95% CI 1.291, 3.00). *Conclusions*. The study revealed that more than one-third of the pregnant women in Ethiopia were found anemic. Its prevalence varied among regions in which the highest (62.7%) and the lowest (11.9%) were from Somali and Addis Ababa, respectively. Hence, efforts should be made by concerned bodies to intervene in terms of the identified risk factors.

## 1. Introduction

Anemia during pregnancy was defined by the World Health Organization and Center for Disease Control and Prevention (CDC) as a hemoglobin concentration of less than 11 g/dL [1]. Globally, prenatal anemia is observed as an indicator of adverse health and socioeconomic consequences. It impairs physical health, cognitive development, and productivity and reflects the poor economic development of a country [2, 3].

Globally, an estimated 32.4 million (38.2%) pregnant women were anemic. Its burden was high in South East Asia (48.7%) and in Africa (46.3%) [2]. Sub-Saharan Africa took the greatest share, where 17.2 million pregnant women were reported anemic [4].

The causes of anemia during pregnancy in developing countries including Ethiopia were nutritional deficiencies of iron, vitamin B12, folate, and parasitic diseases, such as hookworm and malaria. The virtual input of each of these factors to anemia varies greatly in the community [5].

National nutrition program and micronutrient deficiency prevention and control strategy has been implemented in the country to reduce anemia among pregnant mothers [6, 7]. Despite the various efforts made by the government and other stakeholders, maternal anemia is still a major public health concern [8, 9].

Study findings showed variety of results: Southern Ethiopia (27%) [5]; Arba Minch, Ethiopia (32.8%) [10]; Eastern Tigray, Northern Ethiopia (7.8%) [11]; Gondar, Northern Ethiopia (16.6 %) [12]; North West Ethiopia (30%) [13]; North Shoa Zone, Ethiopia (10%) [14].

Even though pocket studies were conducted in various settings of the country and most shreds of evidence lack consistency and unrepresentative samples, there is scarce community-based evidence showing national representatives. Therefore, this community-based and national-wide study was conducted to assess the determinant factors of anemia among pregnant women. The result of this study will be used for national-level policy making and programming by concerned bodies to intervene and lessen the high burden of the disease.

## 2. Materials and Methods

The data used in this analysis were downloaded from the Demographic and Health Survey (DHS) Program website. Administratively, regions in Ethiopia are divided into zones, and zones, into administrative units, called woreda. The 2016 EDHS was conducted on a nationally representative sample of nine regions and two city administrations of the country. They were subdivided into 68 zones, 817 districts, and 16,253 kebeles (lowest local administrative units of the nation).

The EDHS is a periodical survey with a five-year interval; sometimes, in special cases, the interval is different. The 2016 EDHS is the fourth and the most recent DHS in Ethiopia, following 2000, 2005, and 2011 EDHS surveys.

### 2.1. Study Design

A community-based cross-sectional study design was conducted at the national level as one part of the periodic EDHS. The survey was conducted with nationally representative samples from all of the regions of the country. The details of the sample design and sampling procedure, including the sampling framework and implementation and response rates, are provided in the respective EDHS reports (http://www.measuredhs.com/).

### 2.2. Sampling Procedure

The 2016 EDHS data are by now chosen using a stratified, two-stage cluster design, and the enumeration areas were the sampling units for the first stage. In the first stage, 645 enumeration areas were randomly selected: 202 in urban areas and 443 in rural areas. In the second stage, a fixed number of 28 households per cluster were selected randomly for each enumeration area. 18,008 households were randomly selected, and 16,650 households were eligible and interviewed. Additional information about the methodology of EDHS 2016 can be accessed in the report of the main findings of the survey published [13].

### 2.3. Data Extraction Methods

EDHS 2016 data were downloaded, with permission, from the measure DHS website in SPSS. After a review of the detailed data coding, further data recoding was completed. In the 2016 EDHS dataset, there were 3327 pregnant mothers, of whom 155 pregnant mothers were excluded from the analysis data due to missed hemoglobin data. Information on a wide range of sociodemographic, economic, household, and obstetric characteristics, anemia level, and other indicators were extracted.

### 2.4. Measurement and Operational Definition

Anemia status was determined based on hemoglobin concentration in blood adjusted to the altitude. Anemia was defined as the occurrence of a hemoglobin level of less than 11 g/dL. It was further categorized into mild, moderate, and severe anemia with a hemoglobin range of 10–<11 g/dl, 7–<10 g/dl, and <7 g/dl, respectively.

### 2.5. The Study Population

The study population was randomly selected pregnant mothers who have hemoglobin data in their data record in the archive of EDHS 2016 data.

### 2.6. Variables in the Study

#### 2.6.1. The Outcome Variable

The outcome variable is the anemia status of pregnant mothers.

#### 2.6.2. Covariates Variables

To investigate the determinants of anemia among pregnant mothers, a number of sociodemographic, health, and socioeconomic factors, such as maternal and paternal characteristics, household characteristics, and environmental conditions, were assessed.

### 2.7. Data Analysis

After the data were extracted, they were checked for its completeness and consistency, and we did the preliminary analysis. Data analysis was carried out using STATA version v.14. Sample weights were applied to compensate for the unequal probability of selection between the strata, which has also been geographically defined for nonresponses. A detailed explanation of the weighting procedure can be found in the EDHS methodology report [13]. We used “svy” in STATA v.14 to weight the survey data and perform the analyses.

Descriptive statistics was done to describe the data such as frequencies and percentages. Anemia status was determined based on hemoglobin concentration in blood adjusted to altitude. Adjusted concentration less than 11 g/dl was considered as anemic.

Logistic regression method was used to identify the determinants of anemia. Bivariate analysis was performed to determine the crude association of each covariate variables with the outcome variable (anemia status). Those covariate variables with *P* value less than 0.20 in the bivariate analysis were included in the final multivariable logistic regression analysis to adjust for the confounding variable and to identify the final determinant of anemia among pregnant mothers. We use the backward logistic regression method during the multivariable logistic regression analysis. Before inclusion of predictors to the final logistic regression model, the multicollinearity was checked using VIF<10/tolerance >0.1 for continuous independent variables. The goodness of fit of the final logistic model was tested using Hosmer–Lemeshow test at *P* value of >0.05. Outcome measures have been indicated by odds ratio with 95% confidence interval. Finally, covariate variables with *P* value of< 0.05 in the multivariable logistic regression model were considered as statistically significant variables in the final logistic model.

### 2.8. Ethical Considerations

The study proposal has received an ethical approval from Tigray Health Research Institute, and a formal letter of permission was obtained from measure DHS project website to access the dataset (http://www.measuredhs.com).

## 3. Results

### 3.1. Sociodemographic and Household Characteristics of Pregnant Mothers

Data on 3080 pregnant mothers were included in the final analysis. The mean age was15 years with a SD of ± 1.5. Half of the pregnant mothers were in the age group of 30–39 years. 88.5% of them are rural residents, 76.5% are currently not working, and 75% are illiterate. Around half of the pregnant mothers (43.3%) belong to the poorest wealth index (Table 1). 55.7% have an improved source of drinking water, and 54.6% have an improved type of toilet facility. 84% did not have electricity, and 75.3% have no radio. Around half of the households have 4–6 children (Table 2).

### 3.2. Obstetric Characteristics of Pregnant Mothers

Around half of the pregnant women (46.6%) did not follow the ANC, two thirds (62.3%) did not receive iron during pregnancy, and 62.5% received iron for 1–30 days. More than three fourth (88.5%) have never had a terminated pregnancy, 93.4% have not received a drug for intestinal parasite, and 92% had a postnatal checkup (Table 3).

### 3.3. Anemia Prevalence and Level and Regional Distribution among Pregnant Mothers

**Figure 1:**
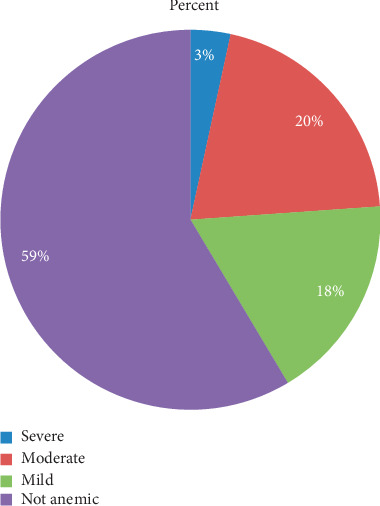
Anemia level among pregnant mothers, EDHS 2016 (*n* = 3082).

**Figure 2:**
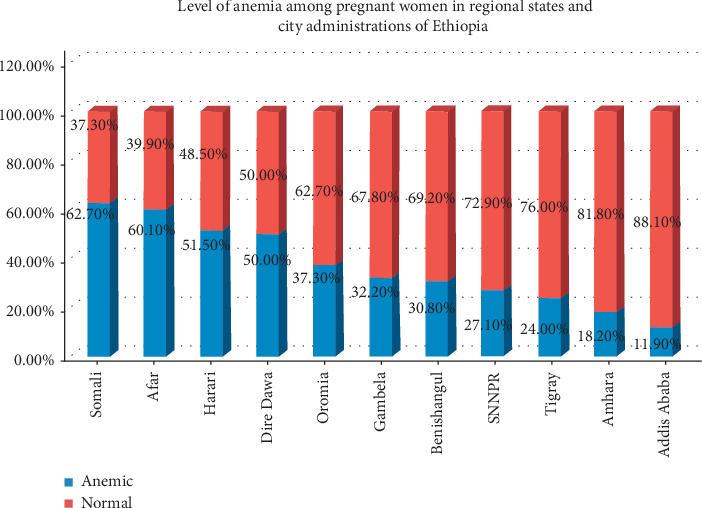
Regional distribution of anemia among pregnant mothers (*n* = 3082).

The prevalence of anemia among pregnant women was 41%. Twenty percent had moderate anemia, 18%, mild anemia, and 3%, severe anemia (Figure 1). There was a wide regional variation of anemia level among pregnant mothers in Ethiopia. The highest anemia level (62.7%) was found in Somali regional state and the lowest in Addis Ababa (11.9 %) (Figure 2).

### 3.4. Factors Associated with Anemia among Pregnant Mothers

As shown in Table 4, the binary logistic regression of mother's age, residence, maternal educational status, religion, number of household members, number of under-five children, sex of household head, having a bank account, wealth index, if the current pregnancy is wanted, having a terminated pregnancy, age at first sexual intercourse, husband's desire for more children, and if the mother is currently working were significantly associated.

The maternal age of 30–39 years was significantly associated with anemia (adjusted odds ratio (AOR) = 1.72 (1.24, 2.39)) compared with an age of less than 20 years. Receiving no education was significantly associated with anemia (adjusted odds ratio (AOR) = 2.19 (1.45, 2.49)). Being a Muslim was significantly associated with anemia (adjusted odds ratio (AOR) = 2.12 (1.69, 2.65)) compared with being Orthodox. The presence of 4–6 members in a household was significantly associated with anemia (adjusted odds ratio (AOR) = 1.44 (1.05, 1.97)) compared with a household having 1–3 members

The presence of 1–3 under-five children in a household was significantly associated with anemia (adjusted odds ratio (AOR) =  1.47 (1.10, 1.97)) compared with having no under-five children. A household head being a female was significantly associated with anemia (adjusted odds ratio (AOR)  =  2.02 (1.61, 2.54)) compared with a male household head. Belonging to the poorest wealth index was significantly associated with anemia (adjusted odds ratio (AOR) = 1.29 (1.22, 1.62)) compared with the richest wealth index. Wanting the current pregnancy (adjusted odds ratio (AOR) = 1.93 (1.44, 2.60)) and wishing the current pregnancy happened later (adjusted odds ratio (AOR)  =  1.75 (1.23, 2.49)) were significantly associated with anemia.

Not having a terminated pregnancy was significantly associated with anemia (adjusted odds ratio (AOR) = 1.49 (1.15, 1.93)) compared with having a terminated pregnancy. An age of 13–17 years (adjusted odds ratio (AOR) = 1.97 (1.29, 3.00)) and ≥18 years (adjusted odds ratio (AOR) = 1.59 (1.02, 2.49)) at the first sexual intercourse were significantly associated with anemia compared with an age of 13 years at the first sexual intercourse (AOR = 1.29; 95% CI (1.014, 1.60)).

## 4. Discussion

Anemia increases the risk of maternal and child morbidity and mortality. It also impairs cognitive and physical development of children and decreases work efficiency in adults [15]. This study showed that the prevalence of anemia among pregnant women was 41% of which 20% were moderately anemic; 18%, mildly anemic; 3%, severely anemic. Identified factors were older age (30–39 years), being illiterate, belonging to the poorest wealth quintile, number of household members being 4–6, number of under-five children being two, household head being a female, current pregnancy wanted later, no terminated pregnancy, being a Muslim, and an age of 13–17 years at first sexual intercourse.

The magnitude of anemia in this study was 41%. The results of this study are consistent with the studies done in Mangalore (41.5%) [16] and Northern Gana (42.7%) [17], while they show a lower prevalence compared with a study conducted in the field practice area of a medical college (81.8%) [18] and a nationally representative survey in India (62.6%).

On the other hand, the results of this study show a higher prevalence compared with study in North West Ethiopia (30.5%) [13]. This could be explained by an extra demand for iron by pregnant women for fetal growth and development during pregnancy and an inability to fulfill the required demand. These differences in the prevalence of anemia might be due to variations in socioeconomic status, attention given for focused antenatal care and supplementation of iron sulfate throughout the pregnancy, dietary patterns, sample size, and geographical and lifestyle variations.

In the present study, noneducated pregnant women were significantly associated with anemia. This finding is consistent with previous reports [18, 20, 21]. The possible reason for this might be the fact that educated pregnant women have a better income and knowledge that in turn help them to follow better lifestyle behaviors like consuming iron-rich nutritious foods and better health-seeking habits like the intake of sufficient iron tablets during antenatal care.

Another finding of this study is that being pregnant with an age of 30–39 years was significantly associated with anemia. This is consistent with previous reports [18, 19]. This might be because as maternal age increases, the mother may face repetitive and relentless pregnancies, endure the collective result of the exhausting labor-related complications, and be subjected to other illnesses which may predispose her for anemia.

The head of the household being a female was significantly associated with anemia during pregnancy. According to EDHS 2016 report in Ethiopia, the majority of households' heads were males [22]. Females have limited control over the materialistic and social resources in Ethiopia. Food and nutrients are allocated inequitably within the households with an obvious male benefit.

Male household heads consume more animal-source foods, eat special foods and drink alcohol outside of their home, and have an uppermost dietary adequacy, whereas women eat more low-status foods and have lower dietary adequacy, particularly pregnant women due to their high requirements.

In addition to this, most of the time males are also responsible for paying for health care expenses. The number of household members being 4–6 is significantly associated with anemia during pregnancy. This is in line with the study conducted in India, Gamo Gofa Zone, and Oromia region, Ethiopia [10,23,24]. Since pregnant mothers consume a larger amount of food, food insecurity is one of the underlying causes of anemia, and food-insecure households were at increased risk of pregnancy anemia.

An increased number of children in the household are significantly associated with prenatal anemia. This is also in line with a study in Eastern and Northern Ethiopia and Mangalore as the result showed that unplanned pregnancies, number of pregnancies, and increased number of children have a negative effect on prenatal anemia [16, 25, 26]. This might be due to the association of anemia in pregnancy with food insecurity with the increased number of under-five children.

Pregnant women with the poorest economic status were at the highest risk of anemia, when compared with their wealthy counterpart. This finding was similar to reports in developing countries like Ethiopia, Uganda, and South Asia. [27–29]. This might be due to the fact that having a low income would mean having less money to buy iron-containing nutritious foods or have a balanced diet. As a result, it leads to an insufficient diet and anemic and poor health outcomes.

This study showed that pregnant women who had a terminated pregnancy before were likely to have anemia compared to women who did not have one. In contrast with reports in Adigrate and Bahir Dar, Ethiopia, and teaching Hospital of Mangalor [16, 30, 31], we could not find any report supporting this finding and therefore further study is needed.

### 4.1. Strength and Limitation of the Study

This study used large population-based data with a large sample size, which is representative of all regions of Ethiopia. These findings will present useful information for policy makers to decrease the prevalence of anemia during pregnancy in all regions of the country.

This study made use of cross-sectional data from 2016 Ethiopian Demographic and Health Survey, which does not allow the identification of the precedence in time between exposure and outcome (chicken egg dilemma). Furthermore, EDHS was a questionnaire-based survey and relied on the memory of the respondents, and as such, recall bias in the results might be a weakness for this study.

## 5. Conclusion

More than one-third of the pregnant women in Ethiopia were anemic. Regions with high anemia prevalence among pregnant women should be given due consideration. The factors associated with anemia in this study population were maternal age, belonging to the poorest wealth index, being a Muslim, number of household members being four to six, number of under-five children being two, the head of the household being a female, the current pregnancy wanted later, no terminated pregnancy, and an age of 13–17 years at the first sexual intercourse. The observed prevalence of anemia might affect the birth outcome of pregnant women. The concerned bodies should focus on enhancing and emphasizing the interventions targeting the identified factors to reduce the high prevalence of anemia among pregnant women in the country.

## Figures and Tables

**Table 1:** Sociodemographic and other characteristics of pregnant mothers, EDHS 2016 (*n* = 3082).

Variables	Frequency	Percentage (%)
Maternal age (years)		
<20 years	31	1
20–29	1202	39
30–39	1584	51.4
40–49	265	8.6

Residence		
Rural	2723	88.4
Urban	359	11.6

Mother's educational level		
Illiterate	2313	75
Primary	616	20
Secondary	106	3.4
Higher	47	1.6

Currently working		
Yes	723	23.5
No	2359	76.5

Wealth index combined		
Poorest	1347	43.7
Poorer	534	17.3
Middle	423	13.7
Richer	423	13.7
Richest	355	11.5

**Table 2:** Household characteristics of pregnant mothers, EDHS 2016 (*n* = 3082).

Variables	Frequency	Percentage
Sources of drinking water		
Improved	1714	55.7
Nonimproved	1336	43.3
Others	32	1

Type of toilet facility		
Improved	1682	54.6
Nonimproved	1400	45.4

Having electricity		
Yes	464	15.
No	2588	84
Not a de jure residence	30	1

Having a radio		
Yes	730	23.7
No	2322	75.3
Not a de jure residence	30	1

Having a television		
Yes	203	6.6
No	2849	92.4
Not a de jure residence	30	1

Number of household members		
1–3	287	9.9
4–6	1405	48.3
7–9	1142	39.9
≥10	72	2.5

**Table 3:** Obstetric characteristics of pregnant mothers, EDHS 2016 (*n* = 3082).

Variables	Frequency	Percentage
ANC visit		
No ANC visit	344	46.6
1–3	213	28.7
≥4	185	24.9

Receiving iron during pregnancy		
Yes	277	37.3
No	462	62.3

Days of iron given		
1–30	170	62.5
31–60	45	16.5
>60	57	21

Ever having a terminated pregnancy		
Yes	355	11.5
No	2727	88.5

Drug for intestinal parasite		
Yes	45	6.1
No	693	93.4

Postnatal checkup		
Yes	59	8
No	683	92

**Table 4:** Parameter estimates of related covariates in the final proportional odds model of pregnant mothers, EDHS 2016 (*n* = 3082).

Variables		Anemia		COR (95% CI)	*P* value	AOR (95% CI)	*P* value
		Yes, *n* (%)	No, *n* (%)				
Maternal age	<20 years	12 (38.7)	19 (61.3)	2.068 (0.951, 4.496)	0.067	0.847 (0.343, 2.094)	0.719
	20–29	495 (41.2)	707 (58.8)	2.292 (1.687, 3.115)	<0.001^*∗*^	1.133 (0.790, 1.623)	0.498
	30–39	708 (44.7)	876 (55.3)	2.646 (1.958, 3.576)	<0.001^*∗*^	1.725 (1.241, 2.397)	0.001^*∗*^
	40–49	62 (23.4)	203 (76.6)	1		1	

Residence	Urban	144 (40.1)	215 (59.9)	1			
	Rural	1133 (41.6)	1590 (58.4)	1.064 (0.850, 1.332)	0.588		

Maternal educational status	No education	1022 (44.2%)	1291 (55.8)	2.309 (1.1924.471)	0.013^*∗*^	2.19(1.45, 2.49)	0.01^*∗*^
	Primary	201 (32.6)	415 (67.4)	1.413 (0.718, 2.780)	0.317	0.845 (0.393, 1.817)	0.666
	Secondary	42 (39.6)	64 (60.4)	1.914 (0.893, 4.103)	0.095^*∗*^	1.314 (0.576, 2.999)	0.516
	Higher	12 (25.5)	35 (74.5)	1		1	

Religion	Orthodox	175 (25.7)	505 (74.3)	1		1	
	Protestant	141 (29.4)	338 (70.6)	1.204 (0.927, 1.563)	0.164	0.997 (0.751, 1.323)	0.982
	Muslim	940 (51.2)	896 (48.8)	3.027 (2.492, 3.678)	<0.001^*∗*^	2.117 (1.693, 2.649)	<0.001^*∗*^
	Others	21 (24.1)	66 (75.9)	0.918 (0.546, 1.545)	0.748	0.722 (0.421, 1.238)	0.237

Number of HH members	1–3	105 (36.6)	182 (63.4)	1		1	
	4–6	641 (45.6)	764 (54.4)	1.454 (1.119, 1.890)	0.005^*∗*^	1.443 (1.055, 1.973)	0.022^*∗*^
	7–9	446 (39.1)	696 (60.9)	1.111 (0.850, 1.452)	0.442	0.937 (0.659, 1.332)	0.716
	≥10	85 (34.3)	163 (65.7)	0.904 (0.633, 1.290)	0.578	0.716 (0.457, 1.122)	0.145

Number of under-five children	0	128 (35.0)	238 (65.0)	1		1	
	1	385 (34.9)	717 (65.1)	0.998 (0.779, 1.279)	0.990	0.953 (0.721, 1.260)	0.738
	2	611 (48.5)	648 (51.5)	1.753 (1.377, 2.232)	<0.001^*∗*^	1.471 (1.100, 1.966)	0.009^*∗*^
	≥3	153 (43.1)	202 (56.9)	1.408 (1.043, 1.902)	0.026^*∗*^	0.988 (0.690, 1.415)	0.949

Sex of HH head	Male	988 (38.1)	1604 (61.9)	1		1	
	Female	298 (59.0)	201 (41.0)	2.334 (1.917, 2.842)	<0.001^*∗*^	2.025 (1.614, 2.540)	<0.001^*∗*^

Having a bank account	Yes	40 (19.6)	164 (80.4)	1		1	
	No	1237 (43.0)	1641 (57.0)	3.091 (2.170, 4.401)	<0.001^*∗*^	2.049 (1.376, 3.051)	>0.001^*∗*^

Wealth index	Poorest	684 (50.8)	663 (49.2)	2.153 (1.682, 2.755)	<0.001^*∗*^	1.29 (1.223, 1.643)	>0.001^*∗*^
	Poorer	206 (38.6)	328 (61.4)	1.311 (0.988, 1.738)	0.060^*∗*^	0.926 (0.671, 1.279)	0.641
	Middle	123 (29.1)	300 (70.9)	0.856 (0.630, 1.162)	0.318	0.666 (0.475, 1.735)	0.719^*∗*^
	Richer	149 (35.2)	274 (64.8)	1.135 6(0.842, 1.530)	0.406	0.970 (0.697, 1.351)	0.859
	Richest	115 (32.4)	240 (67.6)	1		1	

Current pregnancy wanted	Then	1027 (44.2)	1295 (55.8)	2.370 (1.829, 3.070)	<0.001^*∗*^	1.934 (1.439, 2.600)	<0.001^*∗*^
	Later	165 (39.2)	256 (60.8)	1.926 (1.407, 2.637)	<0.001^*∗*^	1.756 (1.236, 2.495)	0.002^*∗*^
	Not at all	85 (25.1)	254 (74.9)	1		1	

Ever had terminated pregnancy	Yes	113 (31.8)	242 (68.2)	1		1	
	No	1164 (42.7)	1563 (57.3)	1.595 (1.260, 2.019)	<0.001^*∗*^	1.489 (1.147, 1.935)	0.003^*∗*^

Age at first sexual intercourse	<13 years	35 (23.3)	115 (76.7)	1		1	
	13–17 years	880 (42.8)	1175 (57.2)	2.461 (1.669, 3.628)	<0.001^*∗*^	1.966 (1.286, 3.004)	0.002^*∗∗*^
	≥18 years	362 (41.3)	515 (58.7)	2.310 (1.546, 3.451)	<0.001^*∗*^	1.596 (1.023, 2.489)	0.039^*∗*^

Husband's desire for more children	Both want the same	396 (40.8)	574 (59.2)	1		1	
	Husband wants more	536 (49.4)	550 (50.6)	1.413 (1.186, 1.682)	<0.001^*∗*^	1.093 (0.901, 1.326)	0.368
	Husband wants fewer	67 (34.5)	127 (65.5)	0.765 (0.554, 1.056)	0.103^*∗*^	0.924 (0.652, 1.309)	0.656
	Do not know	272 (34.1)	526 (65.1)	0.750 (0.617, 0.910)	0.004^*∗*^	0.629 (0.507, 0.780)	<0.001^*∗*^

Respondent currently working	Yes	302 (41.8)	421 (58.2)	1			
	No	975 (41.3)	1384 (58.7)	0.982 (0.829, 1.163)	0.834		

## Data Availability

The database and study materials are available upon request directly to the authors.
